# Third-child fertility intention and its socioeconomic factors among women aged 20–34 years in China

**DOI:** 10.1186/s12889-023-15719-3

**Published:** 2023-05-04

**Authors:** Hanmo Yang, Runlin Han, Zhenjie Wang

**Affiliations:** 1 T. H. Chan School of Public Health, Harvard University, 677 Huntington Ave, Boston, MA 02115 USA; 2 Institute of World Economics and Politics, Chinese Academy of Social Sciences, Beijing, 100732 People’s Republic of China; 3 Institute of Population Research, Peking University, No. 5 Yiheyuan Road, Haidian District, Beijing, 100871 People’s Republic of China

**Keywords:** Fertility intention, Influencing factors, Three-child policy, Childcare services

## Abstract

**Background:**

The low birth rates and rapid population aging has drawn considerable attention from scholars and policymakers in China and around the world. In 2021, China launched the policy and supportive measures that allow up to 3 children per couple. This study aims to explore the influencing factors of the third-child fertility intention among women aged 20–34 years in China.

**Methods:**

We draw data from the National Fertility Survey conducted in 2017. The nationally representative survey adopts a stratified, 3-stage, and probabilities proportional to size sampling method. A total of 61,588 valid samples aged 20–34 years old were obtained. Fertility desire and behavior, childbearing and service use, and potential influencing factors of fertility intention such as the history of pregnancy were assessed.

**Results:**

In general, 5.01% of Chinese women of prime childbearing age had fertility intention for a third child, and the proportion varies by region across mainland China. Individual characteristics such as being ethnic minorities, being rural residents, and having more siblings are significantly positively correlated with the third-child fertility intention, while the intention was significantly lower among women with a higher income or education level, migrant women, and those engaged in the non-agricultural labor force. Women who already had a son had lower fertility intention for a third child. Moreover, it was the perceived acceptable costs of childcare services rather than the actual costs that mattered more for the fertility intention.

**Conclusions:**

Our study concludes a series of socioeconomic factors, and previous childbearing and childrearing experiences are crucial for women’s fertility intention for a third child. These findings highlight the importance of launching supportive measures in addition to the introduction of the 3-child policy in promoting a fertility-friendly society.

**Supplementary Information:**

The online version contains supplementary material available at 10.1186/s12889-023-15719-3.

## Background

The low birth rates and rapid population aging has drawn considerable attention from scholars and policymakers in China and around the world. Recent census data in 2020 reveals that the population aged 65 and above has surged to 191 million, accounting for 13.52% of China’s total population, up from 8.92% in 2010 [1]. Meanwhile, the total fertility rate (TFR) was down to 1.30, which is well below the replacement level of 2.1 and the 1.5 warning line of the low fertility trap. These dynamic shifts in the population structure pose a series of challenges to China’s economic development and population wellbeing, such as a shrinking labor force and mounting pressure on social welfare programs.

Multiple survey results have concluded that the ideal number of children for the majority of the Chinese population of childbearing age is usually 2, while the proportion of women with fertility intention for a third child is less than 10%, and the number is even lower for young people below 30 years old [2, 3]. To improve population structure in the long run, China launched the policy and supportive measures that allow up to 3 children per couple (“3-child policy” hereafter) in May 2021.

Despite the policy efforts, fertility intention and behavior should not be expected to boost shortly, since many other socioeconomic factors may have greater impacts [4]. At the individual level, various factors such as educational attainment, employment status, and income can have a mixed impact on women’s fertility intentions. While a higher socioeconomic status can increase the opportunity cost of childbearing, it can also provide greater financial security [5–7]. At the family level, access to financial and childcare support from family members plays a crucial role in shaping women’s desire to have children [8]. At the societal level, cultural norms, the social security system, and access to reproductive healthcare services can all influence individuals’ desires and expectations regarding childbearing [9].

Compared with giving birth to the first child, the marginal utility, i.e. the additional benefits or satisfaction a family perceived for having the second or the third child compared to the first one, could be significantly different for a family [10, 11]. The cost of having more children can also differ as it can put a strain on the family’s finances and emotional resources. As a result, the factors that affect women’s fertility intentions can vary across parity. For example, when considering additional children, financial factors may play a more significant role, whereas relationship status or career goals may be more relevant for first-time parents.

Though the reproduction willingness of families that already have 1 child has been well studied [12–14], less attention has been paid to women’s previous childbearing or childrearing experience as an influencing factor. A positive experience may influence women’s fertility intentions by decreasing the perceived burden of giving birth and improving her perceived ability to balance motherhood with other responsibilities such as career development. Furthermore, numerous studies have carried out heterogeneity analyses on specific groups, such as the rural-to-urban migrant populations [13], the only child families [15], and urban couples [16, 17]. However, few studies have investigated women of prime childbearing age in China. Addressing the specific concerns and needs of women during childbearing and childrearing, especially for women of prim childbearing age, can improve their experience and encourage them to pursue their fertility desires with less concern.

With the high cost and inconsistent quality, childcare services in China are still far from ideal [18, 19]. State-owned enterprises (SOEs) used to provide free or low-cost public childcare services to employees, but the number of such facilities decreased sharply along with the SOE reform in 2000 when the SOEs were privatized [20]. To ease the concern of childrearing, a series of policies were passed since 2010 to encourage the entrance of private kindergartens, but many parents in China struggle to find suitable and reliable childcare services due to unsatisfactory quality and high costs.

Using nationally representative survey data, this study investigates the influencing factors and their marginal impacts on the third-child fertility intention among women aged 20–34 years. We pay special attention to women in the labor force and women with childbearing and childrearing experience. Since the survey was conducted prior to the introduction of the 3-child policy, our research serves as a baseline result for evaluating fertility support measures and contributes to the literature as a comparative reference for future research on related topics using more updated data.

## Method

### Data

We draw data from the National Fertility Survey conducted by the former National Health and Family Planning Commission in 2017. The nationally representative survey adopted a stratified, 3-stage, and probabilities proportional to size sampling method, covering approximately 250,000 women aged 15–60 years from 12,500 villages/communities across all provincial-level administrative units in mainland China. With an aim to better understand the fertility intention of women and the need for fertility-related public services, the questionnaire included questions on fertility desire and behavior, childbearing and service use, and potential influencing factors of fertility intention such as the history of pregnancy.

The survey was conducted through both face-to-face interviews and online questionnaires and was subject to strict quality control measures such as effective management, standardized operational guidelines, comprehensive training, onsite monitoring and technical support, ex post quality checks, and data comparisons with other sources. The sampling design weights were constructed at the national level according to women’s age, marital status, and household registration status. These rigorous measures played a critical role in the successful implementation of the survey and ensured the high quality of the data [21, 22].

In this study, we focus on the fertility intention of women aged between 20 and 34, which is considered the primary childbearing age by the National Health Commission of China and is commonly used in the context of many countries. We exclude the younger age group due to their early stage of reproductive life, which may result in an irrational perception of childbearing. Many women in this age group are still pursuing higher education or starting their careers and lack a stable partner or financial stability. As such, their fertility intentions are subject to various factors that are highly likely to change over time [23]. Although women over 34 years old are also inclined to have the intention of giving birth to a third child, their fertility intentions are more likely to be constrained by health concerns, which is not the focus of this study [24]. By focusing on the 20–34 age range, this study aims to provide a more targeted understanding of the fertility intentions of women who are actively contemplating or planning to have children. A total of 61,588 valid samples were obtained after excluding women who had already given birth to a third child and less than 1% of observations with missing information on key variables.

The current analysis was approved by the Biomedical Ethics Committee of Peking University (IRB00001052-22049). The research was performed in accordance with the Declaration of Helsinki. Informed consent was obtained from all participants during the interview.

### Assessment of fertility intention

The survey collected information on the intended number of children by asking “How many children do you plan to have?”, the answers to which include the number of children already have and children plan to have in the future. We recategorize the answers to generate a binary variable of “intend to have 3 children or more”: 0 if the answer is below 3, i.e., there was no fertility intention for a third child; and 1 if the answer is 3 or above, i.e., there was fertility intention for a third child. We construct the indicator of “ideal number of children is 3 or more” following the same logic, based on the question “How many children do you think is ideal for a family?”. Though the 2 indicators are strongly correlated, the intended number of children are individual’s rational choice based on real conditions, which is a better predictor for the actual fertility behavior [25, 26]. On the other hand, the ideal number of children is a nearly idealized norm, which is subject less to the social, economic, and policy context [22].

### Assessment of influencing factors

To capture the characteristics of the interviewees and their families, we use age, age-squared, number of siblings, marital status (0 = “unmarried (widowed/divorced/never married)”, 1 = “married or cohabiting”), ethnicity (0 = “Han ethnic group”, 1 = “ethnic minority”), migration status (0 = “non-migrant population”, 1 = “migrant population”), and residence area (0 = “urban”, 1= “rural”), as indicators. We assume a curvilinear association between age and fertility intention among women to capture the mixed impact of concerns related to age. For instance, as women age, they may become more financially and psychologically prepared for motherhood, which could lead to a growing desire for children. However, they may also face different social and cultural pressures related to childbearing. Migrant population refers to respondents whose current residing place was different from their registered *hukou* address, many of whom are rural-to-urban migrant workers. Rural and urban residence areas are defined based on the administrative classification of the respondents’ current residing address.

We include the educational and employment statuses of both the women and their spouses: educational status was categorized into 4 groups (0 = “elementary school and below”, 1 = “junior secondary school”, 2 = “senior and technical secondary school”, and 3 = “college and above”); employment status was categorized into 4 groups (0 = “agricultural employment”, 1 = “non-agricultural employment”, 2 = “homemaking”, 3 = “others”). We identified the homeownership status of the current residence as “rented”, “self-built”, or “purchased” and coded 0, 1, and 2, respectively. “Self-built” residence refers to houses that are constructed by the homeowner themselves, which are typically found in rural areas in China and are more affordable options compared to pre-built houses.

For analyses of the fertility intention of women in the labor force, we further include income level and occupation type. The annual individual income of female respondents themselves was divided into 4 groups (0 = “10,000 yuan and below”, 1 = “10,001–25,000 yuan”, 2 = “25,001–40,000 yuan”, and 3 = “above 40,000 yuan”), with each group accounting for roughly a quarter of the samples. Occupations were broadly identified as 6 types (0 = “agricultural production and related industry (forestry, animal husbandry, fishery, and water conservancy production) personnel”, 1 = “clerks and related personnel”, 2 = “business and service personnel”, 3 = “production and transportation equipment operators”, 4 = “professional and technical personnel”, and 5 = “personnel in charge of state agencies, enterprises, and institutions”).

For women with at least 1 child, we include an indicator of whether women have already had a son (0 = “no”, 1 = “yes”). To examine the impact of short-term reproductive experience on the willingness to have another child, the survey questions regarding childbirth costs and reimbursement were only posed to those respondents with a child under 1 year old. We include whether they received free folic acid before or during pregnancy (0 = “no”, 1 = “yes”) and calculated the proportion of reimbursement of childbirth cost for women. Additionally, questions pertaining to the actual and acceptable costs for nurseries and kindergartens were only directed toward respondents with a child under 6 years old. We employ the ratio of nursery/kindergarten fees deemed acceptable by mothers to household income as a proxy for the “acceptable cost of childcare services”, and we use the ratio of actual nursery/kindergarten fees to household income as a proxy for the “actual cost of childcare services”.

### Statistical analysis

We first presented the geographical distribution of the proportion of women aged 20–34 years with the third-child fertility intention at the provincial level. We compared women of prime childbearing age with or without the intention, illustrated the distribution of characteristics, and conducted t-tests and chi-square tests. Weighted linear probability model analyses were conducted for women and the married or cohabitating subsample to estimate the marginal effects of the influencing factors and their 95% confidence intervals (95% CIs). To further analyze the influencing factors for women in the labor force, we repeated the exercise and include individual income levels and occupation types. We also paid special attention to those who have already had 1 or 2 children and evaluated the marginal impact of their childbearing and childrearing experience on their third-child fertility intention. Statistical analyses were performed using Stata 16.

## Results

### Geographical distribution of the third-child fertility intention

Table 1 presented the distribution of the third-child fertility intention across China. In general, 5.01% of Chinese women aged 20–34 years intended to have 3 or more children. Women living in western China had a higher fertility intention on average (6.8%), and the proportion was lower in central (4.3%), eastern (5.0%), and northeastern (0.8%) China, especially in the most economically developed cities such as Beijing (1.5%), Tianjin (1.2%), and Shanghai (0.2%).

**Table 1:** Proportion of Women of Prime Childbearing Age with the Intention for a Third Child

	Provinces/provincial level administrative units	Intended number of children ≥ 3 (%)	Ideal number of children ≥ 3 (%)
		(1)	(2)
Western region	Xinjiang	20.87	21.36
	Tibet	15.63	17.53
	Guangxi	12.22	13.74
	Ningxia	8.18	9.05
	Qinghai	7.74	10.97
	Yunnan	6.11	5.92
	Guizhou	6.11	6.57
	Gansu	4.45	3.35
	Sichuan	3.95	3.49
	Shaanxi	2.50	2.21
	Inner Mongolia	1.95	1.45
	Chongqing	1.62	1.35
	Subtotal	6.76	6.58
Central region	Jiangxi	7.22	8.53
	Henan	6.92	5.77
	Hunan	6.77	6.48
	Anhui	3.08	2.28
	Hubei	2.08	1.58
	Shanxi	2.05	1.77
	Subtotal	4.25	4.64
Eastern region	Hainan	13.24	17.38
	Guangdong	8.92	10.79
	Fujian	4.88	4.88
	Shandong	4.54	3.06
	Hebei	3.54	4.59
	Jiangsu	1.66	1.80
	Zhejiang	1.56	1.53
	Beijing	1.53	2.78
	Tianjin	1.23	1.71
	Shanghai	0.24	1.67
	Subtotal	5.00	4.30
Northeastern region	Jilin	1.07	0.73
	Heilongjiang	0.94	0.75
	Liaoning	0.62	0.79
	Subtotal	0.76	0.86
Total		5.01	4.76

Data source: 2017 National Fertility Sampling Survey Data

### Impact of individual and family characteristics

Table 2 shows the weighted characteristic distribution of the women aged 20–34 years who had not yet given birth to a third child. The average ages of the women and their spouses were approximately 29.6 and 31.8 years, respectively. Among the women respondents, 60.8% were married or cohabiting, 11.9% were ethnic minorities, 20.6% were migrant population, and 49.8% were rural residents, with an average of 2 siblings. More than half of women in the labor market were employed in non-agricultural sectors. Among women who already have at least 1 child, 67.7% have a son. For those with a child under 1 year old, 54.9% received free folic acid before or during pregnancy, and on average, 39.0% of their childbirth costs were reimbursed. For those with a child under 6 years old, the average ratio of actual nursery/kindergarten fees to household income was 18.4%, while the ratio of acceptable fees to household income was 15.4%.

**Table 2:** Weighted Distributions of the Study Sample

	All	Third-child fertility intention		Difference test
		No	Yes	
	(1)	(2)	(3)	(4)
N	61,588	58,656	2932	
%				${\chi }^{2}$test
Marital status				537.115^***^
Married	60.75	59.83	83.46	
Unmarried	39.25	40.17	16.54	
Ethnicity				718.225^***^
Ethnic minority	11.87	11.17	29.26	
Han ethnic group	88.13	88.83	70.74	
Migration status				157.445^***^
Migrant population	20.55	20.96	10.38	
Non-migrant population	79.45	79.04	89.62	
Residence area				659.325^***^
Rural	49.79	48.75	75.55	
Urban	50.21	51.25	24.45	
Education level				1300.131^***^
Primary school and below	7.51	6.94	21.58	
Junior secondary school	32.35	3.17	48.41	
Senior or technical secondary school	20.86	21.03	16.72	
College and above	39.28	40.33	13.33	
Spouse’s education level^a^				1149.076^***^
Elementary school and below	4.91	4.58	13.02	
Junior high school	27.08	26.28	4.69	
Senior or technical secondary school	12.86	12.73	16.03	
College and above	55.15	56.41	24.05	
Employment status				957.701^***^
Agricultural employment	14.98	14.35	30.62	
Non-agricultural employment	51.02	51.97	27.43	
Homemaking	21.21	20.66	34.86	
Others	12.79	13.02	7.09	
Spouse’s employment status^a^				810.270^***^
Agricultural employment	11.05	10.42	26.57	
Non-agricultural employment	45.27	45.07	50.08	
Homemaking	0.38	0.37	0.77	
Others	43.30	44.14	43.30	
Homeownership				844.489^***^
Rented	27.29	27.76	15.62	
Self-built	45.25	44.09	74.05	
Purchased	27.46	28.15	10.33	
Annual individual income^b^				709.938^***^
10,000 yuan and below	24.92	23.80	55.40	
10,001–25,000 yuan	23.08	23.28	17.45	
25,001–40,000 yuan	27.06	27.51	14.60	
Above 40,000 yuan	24.95	25.40	12.56	
Occupation type^b^				1073.301^***^
Agricultural production and related industry personnel	18.02	16.79	51.95	
Clerks and related personnel	14.46	14.82	4.48	
Business and service personnel	37.59	38.00	26.24	
Production and transportation equipment operators	9.90	9.95	8.43	
Professional and technical personnel	17.25	17.59	7.93	
In charge of state agencies, enterprises, and institutions	2.79	2.85	0.98	
Having a son^c^				292.871^***^
No	32.31	31.39	47.93	
Yes	67.69	68.61	52.07	
Received free folic acid^d^				
No	45.08	45.45	40.13	6.092^**^
Yes	54.92	54.55	59.87	
Mean (s.d.)				t-test
Age	29.62 (3.33)	29.68 (3.31)	28.62 (3.55)	0.42^***^
Spouse’s age^a^	31.83 (4.41)	31.88 (4.40)	30.93 (4.48)	1.06^***^
Number of siblings	2.10 (1.03)	2.07 (1.00)	2.69 (1.33)	-0.75^***^
Proportion of reimbursement of childbirth costs^d^	38.97 (28.56)	38.65 (28.38)	43.19 (30.51)	-0.037^***^
Actual cost of childcare services^e^	18.41 (21.49)	18.26 (21.04)	20.84 (27.73)	-0.022^***^
Acceptable cost of childcare services^e^	15.35 (16.40)	15.24 (16.02)	17.08 (21.71)	-0.008^**^

Notes: ^*^P < 0.10, ^**^P < 0.05, ^***^P < 0.01. ^a^ Spouse’s age, education level, and employment status are only for the 47,640 married women. ^b^ Annual individual income and occupation type are only for the 38,342 women in the labor force. ^c^ Having a son is only for the 44,450 women with children. ^d^ Received free folic acid and proportion of reimbursement of childbirth costs are only for the 8,042 women with a child under 1 year old. ^e^ Actual cost of childcare services and acceptable cost of childcare services are only for the 33,347 women with a child under 6 years old

The composition of women with the third-child fertility intention was different from that of the other group in many terms: they were comparatively younger and had more siblings, and a larger proportion of them were married or cohabiting, ethnic minorities, and rural residents. On the other hand, a lower proportion of them were migrant population or living in a rented residence. Their education level and individual income level were lower, and the patterns are similar for their spouse. Among women with childbearing and childcaring experiences, those with third-child fertility intention have a higher proportion ever received free folic acid, and on average, their proportion of childbirth costs reimbursed, actual cost of childcare services, and acceptable cost of childcare services are higher as well. The t-test and chi-square test results in column (4) indicate that the differences were statistically significant.

The regression results of individual and family characteristics are shown in Table 3. For all the subjects of our analysis, having an older age ($\beta$ =0.017, 95% CI: 0.012 to 0.022), being ethnic minorities ($\beta$ =0.023, 95% CI: 0.017 to 0.030), being rural residents ($\beta$ =0.007, 95% CI: 0.003 to 0.011), and having more siblings ($\beta$ =0.009, 95% CI: 0.007 to 0.012) increased the probability of intending to have 3 children or more, while being a migrant ($\beta$ =-0.008, 95% CI: -0.013 to -0.004) decreased the probability. From the perspective of socioeconomic status, the higher the level of education of women, the lower their third-child fertility intention ($\beta$ from − 0.028 to -0.046). Taking the women in agricultural employment as the reference group, the probability of having a third-child fertility intention was lower by 2.1% points (95% CI: -0.027 to -0.015) for women employed in non-agricultural sectors. Compared with those living in a rented residence, women living in a self-built ($\beta$ =0.011, 95% CI: 0.006 to 0.016) or a purchased residence ($\beta$ =0.011, 95% CI: 0.006 to 0.015) were more likely to have a third-child fertility intention.

**Table 3:** Coefficients from Weighted Linear Probability Model Regression Results of the Influencing Factors

	All	Married	Married with unaffected fertility intention
	(1)	(2)	(3)
Age	0.017 (0.012 to 0.022) ^***^	-0.007 (-0.017 to 0.004)	0.013 (-0.017 to 0.043)
Ethnic minorities (Ref: Han ethnic group)	0.023 (0.017 to 0.030) ^***^	0.029 (0.020 to 0.038) ^***^	0.041 (0.015 to 0.068) ^***^
Migrant population (Ref: Non-migrant population)	-0.008 (-0.013 to -0.004) ^***^	-0.010 (-0.016 to -0.003) ^***^	-0.013 (-0.034 to 0.008)
Rural residents (Ref: urban residents)	0.007 (0.003 to 0.011) ^***^	0.007 (0.001 to 0.012) ^***^	0.022 (0.004 to 0.040) ^**^
Number of siblings	0.009 (0.007 to 0.012) ^***^	0.008 (0.005 to 0.011) ^***^	0.022 (0.013 to 0.031) ^***^
Education level (Ref: Elementary school and below)			
Junior high school	-0.028 (-0.037 to -0.019) ^***^	-0.024 (-0.034 to -0.014) ^***^	-0.090 (-0.130 to -0.049) ^***^
Senior or technical secondary school	-0.040 (-0.050 to -0.031) ^***^	-0.033 (-0.044 to -0.021) ^***^	-0.116 (-0.158 to -0.073) ^***^
College and above	-0.046 (-0.055 to -0.037) ^***^	-0.035 (-0.047 to-0.023) ^***^	-0.115 (-0.159 to -0.071) ^***^
Employment status (Ref: Agricultural employment)			
Non-agricultural employment	-0.021 (-0.027 to -0.015) ^***^	-0.018 (-0.028 to -0.009) ^***^	-0.038 (-0.069 to -0.008) ^**^
Homemaking	0.003 (-0.004 to 0.009)	-0.001 (-0.010 to 0.008)	0.008 (-0.022 to 0.038)
Homeownership (Ref: Rented)			
Self-built	0.011 (0.006 to 0.016) ^***^	0.011 (0.004 to 0.018) ^***^	0.024 (-0.000 to 0.048) ^*^
Purchased	0.011 (0.006 to 0.015) ^***^	0.009 (0.002 to 0.015) ^***^	0.025 (0.004 to 0.045) ^**^
Spouse’s age		0.004 (-0.002 to 0.010)	0.017 (0.001 to 0.034) ^**^
Spouse’s education level (Ref: Elementary school and below)			
Junior high school		-0.007 (-0.018 to 0.004)	-0.050 (-0.093 to -0.007) ^**^
Senior or technical secondary school		-0.006 (-0.018 to 0.005)	-0.072 (-0.118 to -0.027) ^***^
College and above		-0.014 (-0.026 to -0.002) ^**^	-0.106 (-0.152 to -0.060) ^***^
Spouse’s employment status (Ref: Agricultural employment)			
Non-agricultural employment		-0.000 (-0.031 to 0.030)	0.000 (-0.027 to 0.027)
Homemaking		-0.001 (-0.013 to 0.010)	0.006 (-0.104 to 0.116)
Observations	61,588	47,622	9344
R^2^	0.085	0.101	0.226

Notes: ^*^P < 0.10, ^**^P < 0.05, ^***^P < 0.01. 95% CIs are in parentheses. Age squared of women and the spouse and city fixed effect are controlled

Column (2) reports the regression results for married or cohabiting women, with the characteristics of their spouse controlled. The probability of the respondents having a third-child fertility intention was 1.4% points (95% CI: -0.026 to -0.002) lower if the spouse had a college degree or above than had an education level of primary school or below.

Since the intention to have a third child could be restricted by the universal 2-child policy when the survey was conducted, as a robustness check, we further narrowed down the samples based on the respondent’s answer to the question “Does the universal 2-child policy affect your (re)fertility intention?”. Only those 9,344 married respondents who answered “no” were retained, and we assume that these respondents’ fertility intention would not be affected by the 3-child policy as well. Column (3) shows a consistent result for this subgroup, and the coefficient magnitudes are larger in general. In terms of influencing factors of spouses, the higher the education level of the spouse, the lower the third-child fertility intention for the women ($\beta$ from − 0.050 to -0.106), and spouse employment status had no significant effect on women’s fertility intention.

We further analyze the relative importance of the influencing factors by isolating the contribution of each independent variable to the R-squared of the models [27–29]. The results show that besides the city fixed effect, the most powerful influencing factors are the number of siblings, education level of the respondents and their spouses, employment status, and homeownership (Appendix Table A1).

### Impact of income level and occupation type

To explore the impact of income level and occupation type, the study samples were further limited to women engaged in the labor force. Compared with that for the reference group with an annual individual income of 10,000 yuan or less, the probability of intending to have a third child for women with a higher level of income was significantly lower (Table 4). However, with the increase in income level, the impact gradually weakened ($\beta$ from − 0.021 to -0.010), indicating that the impact of income level on the third-child fertility intention is nonlinear. Compared to women in agricultural production and related industry, those working in other fields were significantly less likely to have a third-child fertility intention ($\beta$ from − 0.032 to -0.041). For the subsample of those whose fertility intention was not affected by the fertility policy, the results remain consistent in general. Results from the relative importance analysis show that occupation type and annual individual income are more influential predictors than ethnicity or homeownership (Appendix Table A2).

**Table 4:** Coefficients of Income and Occupation Type for Women Engaged in the Labor Force

	Married		Married with unaffected fertility intention	
	(1)	(2)	(3)	(4)
Annual individual income (Ref: 10,000 yuan and below)				
10,001–25,000 yuan	-0.021 (-0.027 to -0.015) ^***^		-0.070 (-0.098 to -0.042) ^***^	
25,001–40,000 yuan	-0.017 (-0.023 to -0.011) ^***^		-0.049 (-0.077 to -0.020) ^***^	
Above 40,000 yuan	-0.010 (-0.016 to -0.003) ^***^		-0.052 (-0.080 to -0.023) ^***^	
Occupation type (Ref: Agricultural production and related industry personnel)				
Clerks and related personnel		-0.036 (-0.045 to -0.027) ^***^		-0.064 (-0.097 to -0.031) ^***^
Business and service personnel		-0.032 (-0.040 to -0.024) ^***^		-0.061 (-0.092 to -0.030) ^***^
Production and transportation equipment operators		-0.035 (-0.044 to -0.025) ^***^		-0.057 (-0.096 to -0.017) ^***^
Professional and technical personnel		-0.041 (-0.053 to -0.030) ^***^		-0.068 (-0.101 to -0.034) ^***^
In charge of state agencies, enterprises, and institutions		-0.034 (-0.046 to -0.022) ^***^		
Observations	38,342	38,342	5457	5457
R^2^	0.105	0.106	0.258	0.256

Notes: ^*^P < 0.10, ^**^P < 0.05, ^***^P < 0.01. 95% CIs are in parentheses

Age, age-squared, ethnicity, migration status, urban/rural residence, number of siblings, education level, homeownership, and city fixed effect are controlled. Since women in charge of state agencies, enterprises, and institutions are more affected by the fertility policies, this group was excluded from the samples in columns (3) and (4)

### Impact of childbearing and childrearing experience

To explore the impact of childbearing and childrearing experience, this section focuses on women who have already had at least 1 child. Table 5 column (1) and (4) provide the regression results for the samples living in rural areas, and column (2) and (5) provide the results for those living in urban areas, since their experiences and support received may have different impacts on their fertility intention for rural and urban residents. The results consistently show that having a son significantly reduced the third-child fertility intention, especially for rural residents ($\beta$ from − 0.094 to -0.117). Table 5 Panel A presents the coefficients on reproductive experience for women with a child under 1 year old. When controlling for individual characteristics and city fixed effects, the utilization of supporting childbearing measures does not have a significant effect.

**Table 5:** Coefficients of Childbearing and Childcare Services Utilization Experience for Women with Children

	Rural	Urban	${\chi }^{2}$	Rural	Urban	${\chi }^{2}$
	(1)	(2)	(3)	(4)	(5)	(6)
*Panel A. With a child under 1 year old*						
Having a son	-0.117 (-0.139 to -0.094) ^***^	-0.027 (-0.044 to -0.011) ^***^	42.53^***^			
Proportion of reimbursement of childbirth costs	0.027 (-0.008 to 0.063)	0.009 (-0.015 to 0.033)	0.60			
Received free folic acid	-0.004 (-0.023 to 0.014)	0.012 (-0.003 to 0.028)	2.49			
Observations	4933	3056				
R^2^	0.201	0.145				
*Panel B. With a child under 6 years old*						
Having a son	-0.113 (-0.129 to -0.097) ^***^	-0.035 (-0.046 to -0.023) ^***^	79.94^***^	-0.094 (-0.104 to -0.084) ^***^	-0.027 (-0.034 to -0.020) ^***^	132.80^***^
Actual cost of childcare services	0.013 (-0.017 to 0.042)	-0.012 (-0.043 to 0.019)	1.15			
Acceptable cost of childcare services				-0.028 (-0.051 to -0.005) ^***^	-0.025 (-0.047 to -0.003) ^***^	0.00
Observations	8872	5794		19,872	13,244	
R^2^	0.156	0.110		0.142	0.077	

Notes: ^*^P < 0.10, ^**^P < 0.05, ^***^P < 0.01. 95% CIs are in parentheses. Age, age-squared, ethnicity, migration status, urban/rural residence, number of siblings, education level, homeownership, employment status, and city fixed effect are controlled

Childrearing comprises a significant portion of a family’s expenses and has a considerable impact on their budget and quality of living. Parents develop their childrearing budget based on the household’s actual economic status, and ideally, this cost will not impede their consumption habits and is comfortably affordable. Nonetheless, the actual cost of childcare services, such as nurseries and kindergartens, varies depending on factors like location and quality of facilities and services. The disparity between the actual and acceptable cost may lead to mothers spending more money on raising children than they intended, ultimately impacting their reproductive intentions. Hence, our analysis distinguishes between the impact of the “acceptable cost of childcare services” and the “actual cost of childcare services” of utilizing childcare services.

Table 5 Panel B presents the estimates for women with a child under 6 years old. The regression results show that the proportion of children’s monthly nursery/kindergarten costs to their monthly household income had no significant impact on their fertility intention. Nevertheless, the higher the ratio of the acceptable nursery/kindergarten costs to the income, the lower the third-child fertility intention ($\beta$ from − 0.025 to -0.028).

We examine whether the coefficients differ across rural and urban regions in a statistically significant way with a Wald chi-square test. As shown in column (3) and (6), only the null hypotheses of Wald chi-square tests on the estimates of having a son are rejected, meaning that the impact of having a son on lowering third-child fertility intention is significantly stronger for rural residents than for urban residents, while other childbearing and childcare services utilization experience does not yield a different effect on women living in rural and urban areas. Moreover, results from the relative importance analysis suggest that having a son is the most important influencing factor for third-child fertility intention in our models, while other childbearing and childrearing experiences show less explanatory power (Appendix Table A3).

## Discussion

To our knowledge, this is the first study with a large nationally representative population sample to explore the socioeconomic factors on the third-child fertility intention among women of prime childbearing age in China. Consistent with previous literature, our results suggest that rural residents, ethnic minorities, non-migrant women, and homeowners have stronger intentions for having more than 1 child [30–32]. Due to the traditional son-preference idea, some families tend to have more children to ensure that there is at least 1 son in the family, especially in rural areas [33]. Therefore, already having a son is negatively associated with the intention of having more children [34, 35].

Our findings show that having more siblings increase the intention to have more children, implying the importance of trusting relationships with siblings and their fertility-relevant supportive resources in influencing fertility willingness [36, 37]. However, a larger sibship size may also pose a negative impact on fertility intention due to sibling competition for intergenerational support [38]. Previous evidence suggests that migrant women may experience a sense of marginality and insecurity, which depress their fertility intention [39, 40]. Migration costs also delay marriage and first childbirth and extend the birth interval [41, 42]. Our study further calls for equitable access for migrant families to enhanced supportive measures, so as to alleviate their pressure for childbearing and child raising.

Our findings provide further evidence that women in the non-agricultural labor force have less incentive to raise more children. Since childbearing and parenting inevitably take up a large amount of time and energy, the opportunity cost of having more children for professional women is higher than that for women in homemaking or farming [5]. A growing body of research has shown that mothers pay a significant wage penalty for having children [43–45], not least in China [20, 46, 47]. A similar logic applies to education attainment: women with more years of education tend to postpone marriage and childbirth while pursuing career development and increasing opportunity costs inhibit their fertility intention [5, 6]. Nonetheless, women with a higher education or income level are less constrained financially when making fertility decisions [7]. Therefore, some studies suggest that the relationship between socioeconomic status and fertility intention could be an inverted U shape [48, 49].

The marginal role of fertility policies in guiding fertility behavior has been significantly weakened in China [12, 15, 50–52], while the quantity, quality, and price of resources for childrearing have more visible effects on fertility intention [9]. For women in the labor force, institutional benefits such as maternity allowance and maternity leave directly increase the disposable income and childbearing time for families, which relaxes the budget and time constraint for having more children [53, 54]. On the other hand, the gendered childcare leave policy and discriminatory hiring practices may lead women to view having multiple kids and a successful career as incompatible [55].

Our findings provide evidence that compared with the actual cost, it is the perceived affordability of childcare services that plays a more important role in fertility intention. Women of prime childbearing age attach great importance to the quality of childcare and could repress their fertility intention due to concerns about service costs. This result echoes the quality-quantity tradeoffs proposed by Becker and Lewis in such that parents who value children’s quality raise the cost of having children, thereby reducing fertility intention [56, 57].

The strengths of our study include the large sample size, the population-based design, and the adjustment for a wide range of demographic and socioeconomic characteristics. Several limitations of this study should be mentioned and taken into consideration by future researchers. Our data comes from a survey conducted in 2017, prior to the introduction of the 3-child policy in 2021. The policy and supportive measures may have changed the social norms and child-raising environment, thus the level of fertility intention reflected in the survey may be underestimated. As a cross-sectional study, we cannot deduce causative pathways between socioeconomic factors and fertility intention. Nevertheless, our research serves as a baseline result for evaluating fertility support measures and contributes to the literature as a comparative reference for future research on related topics using more updated data.

## Conclusion

Using nationally representative data from the National Fertility Survey in 2017, we explore the influencing factors and their marginal impacts on the third-child fertility intention among women aged 20–34 years in China. Our findings indicate that individual characteristics such as being ethnic minorities, being rural residents, and having more siblings are significantly positively correlated with the third-child fertility intention, while the intention was significantly lower among women with a higher income or education level, migrant women, and those in the non-agricultural labor force. Further investigation on the impact of childbearing and childrearing experiences showed that women who already had a son had a lower third-child fertility intention. Moreover, it was the perceived affordability of childcare services rather than the actual costs that mattered more for the fertility intention.

To promote long-term balanced development of the population, it is necessary to implement relevant supportive measures to build a fertility-friendly society. Our findings suggest that acceptable childbearing and childcare supportive services with satisfactory quality should cover all individuals, including rural-to-urban migrant workers and other economically disadvantaged groups.

In addition, further efforts should be made to reduce the implicit employment discrimination against professional women due to childbirth. As a series of measures such as providing childcare support at work and flexible parental leave support are being promoted after the launch of the 3-child policy, future research may focus on evaluating how these measures help working women balance their careers and family responsibilities and how it may impact fertility intention.

## Electronic supplementary material

Below is the link to the electronic supplementary material.

Supplementary Material 1

## Data Availability

The datasets analyzed during the current study were provided by the National Health Commission of China for restricted research purposes only and are not publicly available.

## Abbreviations

CI: Confidence interval
