# Supplementary material for: Third-child fertility intention and its socioeconomic factors among women aged 20–34 years in China
Source: BMC Public Health. 2023 May 4;23:821. doi: 10.1186/s12889-023-15719-3 (PMC10161537; doi:10.1186/s12889-023-15719-3)
Supplement: Supplementary file 1 — Supplementary Material 1 [file 12889_2023_15719_MOESM1_ESM.docx]

**­­­Third-Child Fertility Intention and Its Socioeconomic Factors among Women Aged 20-34 Years in China**

**Appendix**

**Table A1. Relative Importance of the Influencing Factors of Third-Child Fertility Intention**

|  | All | | Married | |
| --- | --- | --- | --- | --- |
|  | Respective Contributions  towards R-sq | Rank | Respective Contributions  towards R-sq | Rank |
|  | (1) | (2) | (3) | (4) |
| City fixed effect | 58.41 | 1 | 53.67 | 1 |
| Number of siblings | 10.23 | 2 | 9.91 | 2 |
| Education level | 9.30 | 3 | 8.78 | 3 |
| Spouse’s education level |  |  | 7.63 | 4 |
| Employment status | 7.10 | 4 | 5.46 | 5 |
| Spouse’s employment status |  |  | 1.80 | 9 |
| Ethnicity | 5.42 | 5 | 3.28 | 7 |
| Homeownership | 4.29 | 6 | 3.56 | 6 |
| Urban/rural residence | 3.07 | 7 | 2.83 | 8 |
| Migration status | 0.96 | 8 | 0.82 | 10 |
| Age | 0.60 | 10 | 0.44 | 14 |
| Age-squared | 0.63 | 9 | 0.44 | 13 |
| Spouse’s age |  |  | 0.72 | 11 |
| Spouse’s age-squared |  |  | 0.65 | 12 |

**Table A2. Relative Importance of the Influencing Factors for Women Engaged in the Labor Force**

|  | Respective Contributions  towards R-sq | Rank | Respective Contributions  towards R-sq | Rank |
| --- | --- | --- | --- | --- |
|  | (1) | (2) | (3) | (4) |
| City fixed effect | 56.09 | 1 | 56.51 | 1 |
| Number of siblings | 9.70 | 3 | 9.61 | 3 |
| Education level | 11.59 | 2 | 11.30 | 2 |
| Occupation type |  |  | 8.93 | 4 |
| Annual individual income | 8.49 | 4 |  |  |
| Ethnicity | 5.31 | 5 | 5.24 | 5 |
| Homeownership | 3.89 | 6 | 3.76 | 6 |
| Urban/rural residence | 3.23 | 7 | 3.10 | 7 |
| Migration status | 0.72 | 8 | 0.70 | 8 |
| Age | 0.48 | 10 | 0.42 | 10 |
| Age-squared | 0.50 | 9 | 0.44 | 9 |

|  | With a child under 1 year old | | With a child under 6 years old | | | |
| --- | --- | --- | --- | --- | --- | --- |
|  | Respective Contributions  towards R-sq | Rank | Respective Contributions  towards R-sq | Rank | Respective Contributions  towards R-sq | Rank |
|  | (1) | (2) | (3) | (4) | (5) | (6) |
| City fixed effect | 59.14 | 1 | 62.77 | 1 | 57.41 | 1 |
| Having a son | 11.91 | 2 | 15.46 | 2 | 10.64 | 2 |
| Number of siblings | 7.67 | 3 | 4.13 | 4 | 7.45 | 3 |
| Education level | 5.93 | 4 | 2.18 | 7 | 5.95 | 4 |
| Employment status | 2.62 | 7 | 4.20 | 3 | 4.05 | 6 |
| Ethnicity | 3.04 | 5 | 2.07 | 8 | 4.35 | 5 |
| Homeownership | 2.88 | 6 | 3.80 | 5 | 3.72 | 7 |
| Urban/rural residence | 2.14 | 8 | 2.33 | 6 | 2.52 | 8 |
| Migration status | 0.89 | 11 | 0.78 | 11 | 0.96 | 11 |
| Age | 1.59 | 9 | 1.08 | 9 | 1.47 | 9 |
| Age-squared | 1.47 | 10 | 1.00 | 10 | 1.36 | 10 |
| Proportion of reimbursement  of childbirth costs | 0.67 | 12 |  |  |  |  |
| Received free folic acid | 0.07 | 13 |  |  |  |  |
| Actual cost of childcare services | |  | 0.19 | 12 |  |  |
| Acceptable cost of childcare services | |  |  |  | 0.13 | 12 |

**Table A3. Relative Importance of the Influencing Factors for Women with Children**
